# Aortic valve replacement and prosthesis-patient mismatch in the era of trans-catheter aortic valve implantation

**DOI:** 10.1007/s11748-016-0657-9

**Published:** 2016-05-27

**Authors:** Shigeki Morita

**Affiliations:** Department of Thoracic and Cardiovascular Surgery, Faculty of Medicine, Saga University, 5-1-1 Nabeshima, Saga City, Saga 849-8501 Japan

**Keywords:** Aortic valve replacement, Small aortic root, Aortic root enlargement: trans-catheter aortic valve implantation, Patient-prosthesis mismatch

## Abstract

**Objective:**

The treatment strategy for aortic stenosis (AS) has been changing due to newly developed valvular prostheses and trans-catheter aortic valve implantation (TAVI). To determine the role of new modalities for AS with a small aortic root, papers using the concept of prosthesis-patient mismatch (PPM) were reviewed.

**Methods:**

First, to determine the cut-off value of the indexed effective orifice area (IEOA) for defining PPM, the studies of surgical aortic valve replacement (SAVR) with a follow-up longer than 5 years and a patient number larger than 500 were reviewed. Second, the papers comparing TAVI and SAVR were reviewed. Furthermore, the prevalence of PPM was reviewed, with the addition of papers on aortic root enlargement, sutureless AVR, and aortic valve reconstruction with autologous pericardium.

**Results and conclusion:**

The results of the long-term survival after aortic valve replacement (AVR) have indicated that an IEOA less than 0.65 cm^2^/m^2^ should be avoided in all cases, whereas the indications for patients with an IEOA between 065 and 0.85 cm^2^/m^2^ should be determined by considering multiple factors. A large body size and younger age have a significantly negative influence on the long-term survival. In Asian population, the prevalence of PPM was low, despite the fact that the size of the aortic annulus was small. The IEOA after TAVI was larger than after surgical AVR in population-matched studies. To evaluate the role of TAVI and other modalities for a small aortic root, studies with a longer follow-up and larger volume are thus warranted.

## Introduction

Multiple registries and publications indicate that the number of aortic valve replacements (AVR) is increasing. According to the annual report by The Japanese Association for Thoracic Surgery, the number of isolated AVR cases in Japan (concomitant coronary artery bypass included) increased from 4963 (2003) to 10,034 (2013), a more than 100 % increase during the 10-year period [1, 2]. It is expected that the number will continue to increase because of the increase in an aging population and the increase in comorbidity including hypertension, diabetes, or renal failure requiring chronic hemodialysis. A small aortic root remained a concern, especially in the Asian population because of the patient’s small body size. In the days when the availability of a small sized prosthesis was limited, the procedure of choice for patients with a small aortic annulus was AVR with aortic root enlargement (ARE) [3]. The concept of prosthesis-patient mismatch (PPM), first proposed by Rahimtoola et al. [4] and re-visited by Pibarot et al. [5], provided the logical background to select the proper sized prosthesis with the data of the indexed effective orifice area (IEOA), derived from the EOA of the prosthesis and the body surface area of the patient. Pibarot et al. proposed to avoid an IEOA less than 0.85 cm^2^/m^2^ to prevent PPM. The framework of PPM encouraged the use of prostheses with a larger EOA, such as a stentless bioprosthesis, bioprosthesis made of heterologous pericardium or mechanical prostheses designed for supra-annular implantation. If no prosthesis was available to prevent PPM, then ARE was indicated. Reflecting the current practice of the treatment of AS, we cardiac surgeons are undeniably influenced by the data provided by the concept of PPM. However, how solid is the concept and how is it applied to new treatment strategies? To discuss the issue of the treatment strategy of AS with the guidance of PPM, several questions should be answered.

First, the validity of the concept of PPM should be confirmed. When Pibarot proposed his framework, there was no long-term data available to assess the cut-off point of the IEOA for the definition of PPM. Second, using the validated framework of PPM, selection of the prosthesis or procedure including trans-catheter aortic valve implantation (TAVI) should be discussed. In this paper, I will review the long-term studies of PPM after AVR. Next, with the PPM data reinforced by the long-term results, I would like to discuss and share the result of the current therapeutic options including surgical AVR (SAVR), TAVI, ARE, and aortic valve reconstruction with autologous pericardium.

### Influence of PPM on long-term survival

The concept of prosthesis-patient mismatch (PPM) was first proposed by Rahimtoola et al. in 1978 [4], the era of tilting disc and/or ball-and-cage prostheses. In this paper, Rahimtoola et al. foresaw the consequence of using prosthesis with a small orifice area relative to the body size, which would cause obstruction of the outflow and inflow of the ventricle. The recent widespread prevalence of the concept of PPM has been brought about by the framework proposed by Pibarot et al. [5]. They developed a comprehensive framework based on the relationship between the IEOA and the mean pressure gradient of the aortic valve prostheses. When it was plotted on the X–Y planes, the relationship showed a fairly good fit to an exponential curve. The curve indicated a steep increase in the mean pressure gradient when the IEOA was less than 0.85 cm^2^/m^2^. Using this framework, numerous studies have evaluated the hemodynamic performance of aortic valve prosthesis after AVR. The problem, however, was that the framework only provides mechanical relationship between the IEOA and the mean pressure gradient. When Pibarot et al. proposed the framework; they did not provide its consequence on the long-term survival, a hard clinical end point. Since then, several papers have examined the influence of PPM on a late survival using various cut-off values of IEOA.

Table 1 summarizes the papers describing the long-term results (follow-up longer than 5 years) of aortic valve replacement with the data of the IEOA. Only papers with study volumes larger than 500 patients are listed [6–13].

**Table 1:** Definition of PPM and early and late survivals

	Patient number	PPM definition	Influence of PPM on early survival	Influence of PPM on late survival
Ruel et al. (2006) [12]	805	IEOA < 0.85	N/A	Yes for patients with impaired LVEF
Flameng et al. (2006) [6]	506	IEOA < 0.85	No	No
Walther et al. (2006) [13]	4131	IEOA < 0.85	Yes	Yes in the 8.5-year survival (77 vs 81 %, *p* < 0.01)
Moon et al. (2006) [10]	1400	IEOA < 0.75	No	Yes in the 10-year survival if age <60 years or BSA > 2.1
Rao et al. (2000) [11]	2154	IEOA < 0.75	Yes in 30 day mortality (8 vs 5 %, *p* = 0.027)	Yes in the valve related 12 year survival (75 vs 84 %, *p* = 0.004)
Garcia-Fuster et al. (2007) [8]	747	IEOA < 0.65	No	Yes with cardiac mortality
Howell et al. (2010) [9]	801	IEOA < 0.60	No	No
Florath et al. (2008) [7]	533	IEOA < 0.60	N/A	Yes in the 7-year survival (52 vs 80 %, *p* = 0.009)

Numbers in square brackets indicate the reference number

*IEOA* indexed effective orifice area (cm^2^/m^2^), *PPM* prosthesis-patient mismatch, *LVEF* left ventricular ejection fraction

With the definition of PPM as an IEOA less than 0.85 cm^2^/m^2^, Flameng et al. [6], and Ruel et al. [12] did not observe a difference in the long-term survival in the overall population, except for patients with a reduced left ventricular function [12]. However, Walther et al. [13] showed a significant difference in the 8.5-year survival rate (76.8 vs 81 %) with a large number of patients (4131 patients). They also showed that an IEOA less than 0.85 cm^2^/m^2^ was a significant risk factor for adverse cardiac events.

Rao et al. [11] set the cut-off point of PPM as an IEOA of 0.75 cm^2^/m^2^. They found a significantly worse 12-year actuarial survival of 74 % with PPM compared to the survival rate of 85 % in patients without PPM. Moon et al. [10] also used the value of 0.75 cm^2^/m^2^. Although they could not find a survival difference in the overall population, the authors showed a worse survival if the patient was young (age less than 60 years) or if the patient was large (body surface area larger than 2.1 m^2^).

Several studies have used the cut-off value of IEOA as 0.60 or 0.65 cm^2^/m^2^ to define PPM. Howell et al. [9] found no differences in the survival, however, the number of patients with an IEOA less than 0.65 cm^2^/m^2^ in their series was small (48 patients, 6 % of the total study population), and the follow-up was relatively short (5 years) compared to the other studies. Except for the paper by Howell et al., the remaining two studies [7, 8] using cut-off point of 0.65 cm^2^/m^2^ showed a significantly worse survival in the long-term survival, which appears reasonable because an IEOA less than or equal to 0.60 cm^2^/m^2^ is defined as severe AS in the 2014 AHA/ACC guideline [14].

PPM has been subdivided into severe (IEOA less than 0.65 cm^2^/m^2^) and moderate (IEOA between 0.65 and 0.85 cm^2^/m^2^) [15]. Reviewing the above mentioned studies in Table 1 with long-term results, I presume that it may be useful to subdivide PPM further into severe (IEOA less than 0.65 cm^2^/m^2^), moderate (0.65–0.75 cm^2^/m^2^), and mild (0.75–0.85 cm^2^/m^2^). The studies indicate that severe PPM should be avoided in all cases, whereas in patients with moderate PPM (IEOA: 0.65–0.75 cm^2^/m^2^), the selection of procedure and/or prosthesis should be made by considering several factors, including the body size and age, because an age less than 60 years or body surface area larger than 2.1 m^2^ appears to have strong negative influence on survival. Regarding patients with mild PPM (IEOA: 0.75–0.85), aggressive approaches, such as ARE could be reserved, especially when the patient is fragile or has comorbidities.

### Prevalence of PPM in the era of TAVI

**Fig. 1:**
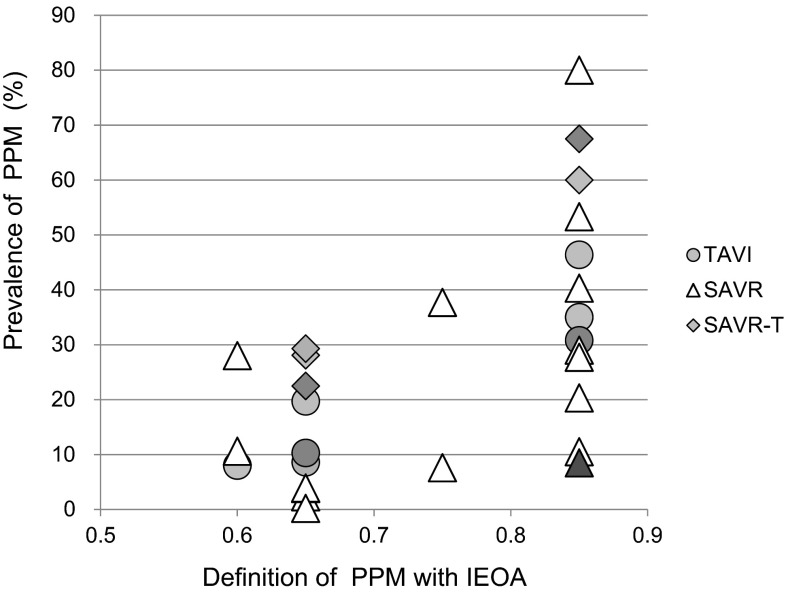
The relationship between the definition of PPM with IEOA and the prevalence of PPM. *Triangles* (SAVR) depict the prevalence reported from the paper studying the SAVR population only. *Circles* (TAVI) represent the prevalence of PPM in patients with TAVI. *Diamonds* (SVAR-T) represent the PPM prevalence of SAVR from the paper comparing TAVI and SAVR. The same *fill color* indicates that the symbols are from the same study. Note that the prevalence is lower in TAVI compared to SAVR. The *triangle* filled in *red* indicates the study of SAVR from the Japanese population Ref. [17], which shows a low prevalence of PPM. Each data point corresponds to the numbers presented in Table 2 (color figure online)

How often do we encounter patients with PPM? In Table 2 and Fig. 1, the prevalence data of PPM reported in the studies are shown [6–13, 16–21]. In addition the data from TAVI are listed. The prevalence of PPM was significantly influenced by the type of prosthesis and by the method of implantation.

**Table 2:** The relationship between IEOA and the prevalence of PPM

	SAVR/TAVI	Patient number	IEOA			
			<0.60	<0.65	<0.75	<0.85
Walther et al. (2006) [13]	SAVR	4131		2.3 %		29.0 %
Rao et al. (2000) [11]	SAVR	2154			7.6 %	10.5 %
Nozohoor et al. (2007) [19]	SAVR	1568		3.8 %		53.3 %
Howell et al. (2010) [9]	SAVR	1418	10.6 %			
Moon et al. (2006) [10]	SAVR	1400			37.7 %	
Ruel et al. (2006) [12]	SAVR	805				40.3 %
Garcia-Fuster et al. (2007) [8]	SAVR	747		3.9 %		27.7 %
Florath et al. (2008) [7]	SAVR	533	28.0 %			80.0 %
Flameng et al. (2006) [6]	SAVR	506		0.2 %		20.2 %
Kaminishi et al. (2013) [17]	SAVR	3609				8.5 %
Takagi and Umemoto (2016) [21]	TAVI	4000	8.0 %			35.1 %
Pibarot et al. (2014) [20]	SAVR	270		28.1 %		60.0 %
Pibarot et al. (2014) [20]	TAVI	304		19.7 %		46.4 %
Giannini et al. (2011) [16]	SAVR	58		29.3 %		
Giannini et al. (2011) [16]	TAVI	58		8.6 %		
Kamperidis et al. (2015) [18]	SAVR*	39		22.5 %		67.5 %
Kamperidis et al. (2015) [18]	TAVI	40		10.3 %		30.8 %

Numbers in square brackets indicate the reference number

*IEOA* indexed effective orifice area (cm^2^/m^2^), *PPM* prosthesis-patient mismatch, *SAVR* surgical aortic valve replacement, *TAVI* trans-catheter aortic valve implantation, SAVR* data from sutureless bioprosthesis

In the sub-study of the PARTNER trial, the prevalence of PPM was compared between TAVI and surgical AVR (SAVR) [20]. The prevalence of PPM was significantly reduced in TAVI compared to SAVR. The reason for the reduced prevalence of PPM in TAVI may be due to the fact that the TAVI device does not have a sewing rim and it can take full advantage of the entire native valve orifice except for the slim thickness of the device wall. Similar results were obtained from other studies comparing SAVR and TAVI. Regarding the prevalence shown in these papers [16, 18, 20, 21], the prevalence of PPM with SAVR was fairly high (Fig. 1; diamonds) compared to the results obtained from paper about SAVR alone (Fig. 1: triangles). The higher prevalence of PPM in these studies may be due to the fact that the SAVR populations were matched with the TAVI populations, and the patient background was different compared with the patients in studies about SAVR only.

Takagi and Umemoto performed a meta-analysis to evaluate PPM in TAVI studies [21]. They showed that the prevalence of PPM after TAVI was 8, 27, and 35 %, for severe PPM (IEOA less than 0.6 cm^2^/m^2^), moderate PPM (IEOA 0.6–0.85 cm^2^/m^2^), and overall PPM (IEOA less than 0.85 cm^2^/m^2^), respectively.

Of note is the paper studying the Japanese population by Kaminishi et al. [17]. The prevalence of PPM was low in Japanese patients (8.5 % for IEOA less than 0.85 cm^2^/m^2^: Fig. 1, red filled triangle). The results indicated that although the annulus diameter of Asian patients is small compared to non-Asian patients, the small body size might have resulted in the low prevalence of PPM. The average body surface area in this study was 1.55 and 1.61 m^2^ with and without PPM, respectively. These values are quite smaller than the average body surface area of 1.87–1.94 m^2^ reported in a study from Europe [13]. In addition, the study period was relatively recent (between January 2008 and December 2009) in the study of Kaminishi et al. Accordingly, currently available pericardial bioprostheses or mechanical prostheses designed for supra-annular implantation were used, which could provide sufficient opening for small sized Asian patients.

### Framework of PPM and currently available modality for AS

Since Pibarot et al. proposed their framework of PPM [5], several new prostheses or procedures have emerged, such as stentless bioprostheses, bioprosthesis using heterologous pericardium, supra-annular type mechanical prostheses, TAVI and sutureless AVR.

The hemodynamic performance and survival results of stentless bioprosthesis were superior to those of porcine aortic bioprostheses [22, 23]. To implant stentless bioprostheses, a sub-coronary technique or full-root technique must be used. Both techniques require a longer ischemic time compared with stented prosthesis. Emergence of stented bioprosthesis using heterologous pericardium and mechanical prosthesis designed for supra-annular implantation reduced the need for stentless bioprosthesis, because the hemodynamic performance was similar among these prostheses.

As mentioned in the previous section, the hemodynamic performance of TAVI was superior to SAVR in a limited patient population. This result, however, cannot be extrapolated to the general population of AS to justify the use of TAVI for patients with small aortic roots. Several issues should be discussed before considering TAVI as an alternative to treat AS patient for small aortic root. Important issues to consider include the long-term results and complications, such as paravalvular leakage, heart conduction disturbance, vascular injury and fatal annulus rupture. A cost benefit analysis of TAVI is also warranted.

In addition, the limitation associated with the usage of the small-size TAVI device, which is related to vascular access, should be mentioned. Currently (as of May, 2016), the smallest TAVI device available in Japan is the 20 mm Sapien XT (Edwards Lifesciences Limited, Irvine, CA, USA), which requires a femoral artery of more than 6 mm in diameter. In most cases, this requirement is not fulfilled in patients who have a small body size. In addition, the 20 mm Sapien XT is indicated for patients with an annulus of between 16 and 19 mm in diameter. Even with the small body size of many Asian patients, few patients have an annulus of between 16 and 19 mm in diameter. Some data indicate that the number of Japanese patients treated with the Sapien XT accounts for less than 10 % of the entire Sapien series. Considering these circumstances, the author assumes that TAVI has not played a major role as a treatment alternative for small aortic annulus.

Although ARE is an established surgical procedure [24], improvements in the hemodynamic performance of the aortic valve prosthesis reduced the need for ARE [3]. Beckamann et al. in Hanover performed a study comparing ARE and SAVR using the sutureless prosthesis. The hemodynamic performance of the implanted prosthesis was similar between the ARE group and the sutureless bioprosthesis group [25]. Implantation of the sutureless bioprosthesis required shorter ischemic and operative times. In this study, the median value of the annulus diameter measured at the time of operation was 19 mm with an average body surface area of 1.8 m^2^, which yielded a projected IEOA of 0.66 cm^2^/m^2^. These data imply that ARE was indicated due to the large body size. In Asian population with small body size ARE may not be indicated with a measured annulus diameter of 19 mm in most of the patients with currently available prostheses.

Ozaki et al. reported a series of aortic valve reconstruction with glutaraldehyde-treated autologous pericardium [26]. The average surgical annular diameter of 416 patients with AS was 20.1 mm. They showed satisfactory hemodynamic results including an average peak pressure gradient of 14.3 mmHg after 5.5 years with a 5-year survival rate of 83.3 %. Because autologous pericardium is sutured to the native aortic annulus, the EOA is expected to include a fully opening valve with the given native annulus. Further studies with a longer follow-up and larger series with multicenter data are surely warranted for this interesting approach.

## Limitations

An important issue regarding the measurement of EOA should be noted as a limitation of this study. In real-world clinical practice, the predicted IEOA is calculated for each patient based on the body surface area of the patient and in vitro EOA data—in most cases, this is obtained from the manufacturer. It is well known that individual postoperative EOA is influenced not only by the type and size of the prosthesis but also by the surgical technique. For example, simple interrupted sutures provide a larger EOA than the supra-annular implantation technique, which uses horizontal mattress sutures [27]. For this reason, it is preferable to collect the postoperative EOA from each patient’s postoperative echocardiography data. The reality is that very few studies have used the postoperative echo data. In the present review only [7] used postoperative echo data, while [8–10] used in vitro data and [6] used intermediate method incorporating a limited number of postoperative echo data. To take advantage of the data provided in this review, it is advisable that each surgeon analyzes the relationship between the EOA provided by the manufacturer and the EOA of their own patients, as determined by postoperative echocardiography.

## Summary and conclusion

An accumulation of data indicates that in patients with AS, postoperative PPM defined by the IEOA influences the long-term survival, especially in patients with a large body surface area and age less than 60 years. Because of the improvement in hemodynamic performance of the currently available prostheses, the prevalence of PPM has been reduced and the need for ARE may be decreasing. Newly developed modalities including TAVI have shown better hemodynamic performance, however, their role in treating small aortic root has yet to be determined. Further study with larger number of patients and longer follow-up is warranted.
